# Poverty and mental health: policy, practice and research implications

**DOI:** 10.1192/bjb.2020.78

**Published:** 2020-10

**Authors:** Lee Knifton, Greig Inglis

**Affiliations:** 1 Centre for Health Policy, University of Strathclyde, Scotland, and Mental Health Foundation, Scotland and Northern Ireland; 2 University of West of Scotland, Paisley

**Keywords:** Poverty, stigma, intersectional stigma, inequality, public health

## Abstract

This article examines the relationship between poverty and mental health problems. We draw on the experience of Glasgow, our home city, which contains some of Western Europe's areas of greatest concentrated poverty and poorest health outcomes. We highlight how mental health problems are related directly to poverty, which in turn underlies wider health inequalities. We then outline implications for psychiatry.

Doctors have often played leading roles in social movements to improve the public's health. These range from the early days of John Snow isolating the role of contaminated water supplies in spreading cholera, through to advocating harm reduction, challenging HIV stigma and, more recently, highlighting the public health catastrophe of mass incarceration in the USA.^1^ Almost all examples are rooted in poverty. There is now increasing recognition that mental health problems form the greatest public health challenge of our time, and that the poor bear the greatest burden of mental illness.^2^

Our article draws on data from Scotland, and especially Glasgow, which contains some of the areas of greatest need and widest health inequalities in Western Europe. However, the relationship between poverty, social stress and mental health problems is not a new phenomenon and was reported by social psychiatrists half a century ago in Langner & Michael's 1963 New York study^3^ and consistently since then. Poverty is both a cause of mental health problems and a consequence. Poverty in childhood and among adults can cause poor mental health through social stresses, stigma and trauma. Equally, mental health problems can lead to impoverishment through loss of employment or underemployment, or fragmentation of social relationships. This vicious cycle is in reality even more complex, as many people with mental health problems move in and out of poverty, living precarious lives.

## Poverty and mental health

The mental health of individuals is shaped by the social, environmental and economic conditions in which they are born, grow, work and age.^4–7^ Poverty and deprivation are key determinants of children's social and behavioural development^8,9^ and adult mental health.^10^ In Scotland, individuals living in the most deprived areas report higher levels of mental ill health and lower levels of well-being than those living in the most affluent areas. In 2018 for example, 23% of men and 26% of women living in the most deprived areas of Scotland reported levels of mental distress indicative of a possible psychiatric disorder, compared with 12 and 16% of men and women living in the least deprived areas.^11^ There is also a clear relationship between area deprivation and suicide in Scotland, with suicides three times more likely in the least than in the most deprived areas.^12^

Inequalities in mental health emerge early in life and become more pronounced throughout childhood. In one cohort study, 7.3% of 4-year-olds in the most deprived areas of Glasgow were rated by their teacher as displaying ‘abnormal’ social, behavioural and emotional difficulties, compared with only 4.1% in the least deprived areas. By age 7, the gap between these groups had widened substantially: 14.7% of children in the most deprived areas were rated as having ‘abnormal’ difficulties, compared with 3.6% of children in the least deprived.^13^ National data from parental ratings of children's behaviour show a similar pattern: at around 4 years of age, 20% of children living in the most deprived areas of Scotland are rated as having ‘borderline’ or ‘abnormal’ levels of difficulties, compared with only 7% living in the least deprived areas.^14^

These findings reflect a broader pattern of socioeconomic inequalities in health that is observed internationally.^15^ The primary causes of these inequalities are structural differences in socioeconomic groups’ access to economic, social and political resources, which in turn affect health through a range of more immediate environmental, psychological and behavioural processes.^16,17^ A wide range of risk factors are more prevalent among low income groups for example, including low levels of perceived control^18^ and unhealthy behaviours such as smoking and low levels of physical activity,^11^ although these are best understood as mechanisms that link the structural causes of inequality to health outcomes.^17^

## Excess mortality and mental health in Glasgow

Glasgow has some of the highest Scottish rates of income deprivation, working-age adults claiming out of work benefits, and children living in low-income families.^19^ Moreover, the city also reports poor mental health, relative to the Scottish average, on a host of indicators, including lower mental well-being and life satisfaction, and higher rates of common mental health problems, prescriptions for anxiety, depression or psychosis, and greater numbers of patients with hospital admissions for psychiatric conditions.^19^

These statistics are consistent with Glasgow's overall health profile and high rates of mortality. Life expectancy in Glasgow is the lowest in Scotland. For example, men and women born in Glasgow in 2016–2018 can expect to live 3.6 and 2.7 fewer years respectively than the Scottish average.^20^ Within Glasgow, men and women living in the most deprived areas of the city can expect to live 13.5 and 10.7 fewer years respectively than those living in the least deprived areas.^21^

The high level of mortality in Glasgow can largely be attributed to the effects of deprivation and poverty in the city, although high levels of *excess* mortality have also been recorded in Glasgow, meaning a significant level of mortality in excess of that which can be explained by deprivation. For example, premature mortality (deaths under 65 years of age) is 30% higher in Glasgow compared with Liverpool and Manchester, despite the similar levels of deprivation between these cities.^22^ Crucially, this excess premature mortality is in large part driven by higher rates of ‘deaths of despair’^23^ in Glasgow, namely deaths from suicide and alcohol- and drug-related causes.^22^

It has been proposed that excess mortality in Glasgow can be explained by a number of historical processes that have rendered the city especially vulnerable to the hazardous effects of deprivation and poverty. These include the lagged effects of historically high levels of deprivation and overcrowding; regional policies that saw industry and sections of the population moved out of Glasgow; the nature of urban change in Glasgow during the post-war period and its effects on living conditions and social connections; and local government responses to UK policies during the 1980s.^24^ On the last point, Walsh and colleagues^24^ describe how the UK government introduced a host of neoliberal policies during this period – including rapid deindustrialisation – that had particularly adverse effects in cities such as Glasgow, Manchester and Liverpool. While Manchester and Liverpool were able to mitigate the negative effects of these national policies to some extent by pursuing urban regeneration and mobilising the political participation of citizens, there were fewer such efforts made in Glasgow, which contributed to the diverging health profiles of the cities.

These researchers have also suggested that this excess mortality may partly reflect an inadequate measurement of deprivation.^24^ However, that does not capture the reality of living in poverty. One aspect of this lived experience that may be important is the experience of poverty-based stigma and discrimination.^25^ Stigma is a fundamental cause of health inequalities,^26^ and international evidence has demonstrated that poverty stigma is associated with poor mental health among low-income groups.^27^ Individuals living in socioeconomically deprived areas may also experience ‘spatial’ stigma, which similarly has a range of adverse health effects for residents^28^ and, crucially, may be unintentionally exacerbated by media and public health professionals’ reports of regional health inequalities.^29^ Given the continued focus on Glasgow's relatively poor health it is possible that the city is more vulnerable to such stigmatising processes. However, we stress that additional research will be required to test whether stigma is an important aspect of the lived reality of poverty, particularly as several psychosocial explanations have already been offered for the excess mortality, with varying levels of supporting evidence.^24^ The notion of intersectional stigma is also gaining traction and requires further research.

Understanding the life-course impact of poverty on mental health is also important. Childhood adversity is one mechanism through which poverty and deprivation have an impact on mental health. Adverse childhood experiences, such as exposure to abuse or household dysfunction, are relatively common in the population. Marryat & Frank examined the prevalence of seven adverse childhood experiences among children born in 2004–2005 in Scotland, and found that approximately two-thirds had experienced at least one adverse experience by age 8.^30^ Moreover, the prevalence was greatest in low-income households: only 1% of children in the highest-income households had four or more adverse childhood experiences, compared with 10.8% in the lowest-income households. Adverse childhood experiences are also strong predictors of mental health in adulthood: individuals who have experienced at least four are at a considerably greater risk of mental ill health, problematic alcohol use and drug misuse.^31^ It has also been suggested that experiences of childhood adversity and complex trauma may contribute to Glasgow's – and Scotland's – excess mortality, particularly that which is attributable to violence, suicide and alcohol and drug-related deaths.^32^ The implications are significant for psychiatry. Not only does it offer a broader explanation of causation; it also highlights the importance of supporting early interventions for young people's mental health and supporting the families – including children – of those experiencing mental health problems.

## Implications

When faced with the scale of the challenge the response can be daunting. This is especially so at a time when we see increasing poverty and socioeconomic inequalities within our society and challenging political conditions. The complexity and enduring nature of the problems necessitate a multilevel response from psychiatry across practice, policy, advocacy and research, which we explore in this section. We argue that this response should address three broad areas.

### Reinvigorate social psychiatry and influence public policy

The demise of social psychiatry in the UK and USA in recent decades has deflected focus away from the social causes and consequences of mental health problems at the very time that social inequalities have been increasing. Now is the time to renew social psychiatry at professional and academic levels. There is considerable scope to form alliances with other areas – especially public mental health agencies and charities. Psychiatry as a profession should support those advocating for progressive public policies to reduce poverty and its impact. If we do not, then, as Phelan and colleagues outline, we will focus only on the intermediate causes of health inequalities, rather than the fundamental causes, and this will ensure that these inequalities persist and are reproduced over time.^33^ Activism with those who have consistently highlighted the links between poverty and mental health problems, such as The Equality Trust, may effect change among policy makers.

### Tackle intersectional stigma and disadvantage

We must understand, research and tackle stigma in a much more sophisticated way by recognising that mental health stigma does not sit in isolation. We need to understand and address what Turan and colleagues define as intersectional stigma.^34^ Intersectional stigma explains the convergence of multiple stigmatised identities that can include ethnicity, gender, sexuality, poverty and health status. This can then magnify the impact on the person's life. In this context, the reality is that you have a much greater chance of getting a mental health problem if you experience poverty. And if you do, then you will likely experience more stigma and discrimination. Its impact on your life will be greater, for example on precarious employment, housing, education and finances. It is harder to recover and the impact on family members may be magnified. Intersectional stigma remains poorly researched and understood,^35^ although the health impact of poverty stigma is now emerging as an important issue in studies in Glasgow and elsewhere.^25^

### Embed poverty-aware practice and commissioning

We conclude with our third idea, to ensure that poverty-aware practice is embedded in services through commissioning, training and teaching. This means that recognising and responding to poverty is part of assessments and care. Income maximisation schemes should be available as an important dimension of healthcare: how to access benefits, manage debt, access local childcare and access support for employment at the earliest stages. This needs to be matched by a major investment in mental health services focused on low-income areas, to address the inverse care law.^36^ These principles are already being put into action. For example across Scotland, including Glasgow, several general practices working in the most deprived areas (referred to as Deep End practices) have recently trialled the integration of money advice workers within primary care, which has generated considerable financial gains for patients.^37^

## About the authors

**Lee Knifton** is Reader and Co-Director of the Centre for Health Policy at the University of Strathclyde, Scotland, and Director of the Mental Health Foundation, Scotland and Northern Ireland. **Greig Inglis** is a lecturer in psychology at the University of West of Scotland, Paisley, Scotland.

## Supplementary material

For supplementary material accompanying this paper visit https://doi.org/10.1192/bjb.2020.78. click here to view supplementary material
